# Adjuvant Effect of Toll-Like Receptor 9 Activation on Cancer Immunotherapy Using Checkpoint Blockade

**DOI:** 10.3389/fimmu.2020.01075

**Published:** 2020-05-29

**Authors:** Yu-Chen Chuang, Jen-Chih Tseng, Li-Rung Huang, Chun-Ming Huang, Chi-Ying F. Huang, Tsung-Hsien Chuang

**Affiliations:** ^1^Immunology Research Center, National Health Research Institutes, Zhunan, Taiwan; ^2^Institute of Molecular and Genomic Medicine, National Health Research Institutes, Zhunan, Taiwan; ^3^Department of Biomedical Sciences and Engineering, National Central University, Taoyuan, Taiwan; ^4^Institute of Biopharmaceutical Sciences, National Yang-Ming University, Taipei, Taiwan

**Keywords:** adjuvant, cancer immunotherapy, CpG-ODN, innate immune, toll-like receptor, immune checkpoint blockade

## Abstract

Immunotherapy using checkpoint blockade has revolutionized cancer treatment, improving patient survival and quality of life. Nevertheless, the clinical outcomes of such immunotherapy are highly heterogeneous between patients. Depending on the cancer type, the patient response rates to this immunotherapy are limited to 20–30%. Based on the mechanism underlying the antitumor immune response, new therapeutic strategies have been designed with the aim of increasing the effectiveness and specificity of the antitumor immune response elicited by checkpoint blockade agents. The activation of toll-like receptor 9 (TLR9) by its synthetic agonists induces the antitumor response within the innate immunity arm, generating adjuvant effects and priming the adaptive immune response elicited by checkpoint blockade during the effector phase of tumor-cell killing. This review first describes the underlying mechanisms of action and current status of monotherapy using TLR9 agonists and immune checkpoint inhibitors for cancer immunotherapy. The rationale for combining these two agents is discussed, and evidence indicating the current status of such combination therapy as a novel cancer treatment strategy is presented.

## Introduction

Major advances have been made in the field of cancer immunotherapy in the past two decades (1, 2). Imiquimod, a toll-like receptor (TLR)7 agonist, was FDA-approved in 1997 under the brand Aldara for treating genital warts and later approved for treating superficial basal cell carcinoma in 2004 (3–5). Three anti-cancer vaccines have been approved by the FDA. BCG (TheraCys), was first approved in 1990 for non-muscle invasive bladder carcinoma (6). Subsequently, Sipuleucel-T (Provenge) was approved for metastatic castration-resistant prostate cancer, and talimogene laherparepvec (T-VEC or Imygic), an oncolytic-virus–based vaccine was approved for advanced melanoma (7, 8). The components of BCG and oncolytic viruses activate TLRs in cells to elicit immune responses (9, 10). Further developments include anti-cancer adoptive cell transfer, including dendritic cell and cytotoxic T-cell therapies, in which patients are treated with *ex vivo* expanded autologous immune cells (11, 12). Studies of T-cell activation and suppression mechanisms have led to the discovery of key checkpoints for immune suppression, including the cytotoxic T-lymphocyte-associated antigen 4 (CTLA-4) (13–15), programmed cell death protein 1 (PD-1), and the PD-1 ligands programmed death-ligand (PD-L)1 and PD-L2 (16–19). The use of antibody (Yervoy, ipilimumab) for immune checkpoint blockade to increase the anti-cancer effect of T-cells was first approved by the FDA in 2011, and several additional checkpoint blockage drugs were subsequently approved (20–22). These immunotherapies have effectively improved the survival and life quality of cancer patients, resulting in their acceptance as the fourth standard treatment for cancers after surgery, chemotherapy, and radiation therapy. In 2016, the American Society of Clinical Oncology (ASCO) announced “Immunotherapy” as the year's top cancer advance. Further, in 2017, the ASCO named “Immunotherapy 2.0” as advance of the year, emphasizing the recent, rapid progress of research into new agents that enhance the innate abilities of immunity to fight cancers (23). Although cancer immunotherapy is a major achievement in fighting cancer, the efficacy for patient treatment is still limited and unsatisfactory. For example, the response rate of patients with solid tumors to checkpoint inhibitors is only 20–30% (24, 25). Therefore, novel strategies to improve the efficacy of cancer immunotherapy are needed.

Cancer cells are targeted by immune surveillance through a process similar to the host immune response to microbe-infected cells. The human immune system is capable of discriminating and destroying cancer cells that display tumor antigens. These tumor antigens originate from self molecules but exhibit antigenic mutations and/or ectopic expression during tumor development (26, 27). Many cellular and molecular factors are involved in this process of immune suppression of tumor growth. Innate immune cells, including natural killer (NK) cells, monocytes/macrophages, and dendritic cells, mediate direct innate antitumor responses and activate adaptive immune cells such as T and B cells to develop memory and long-term responses to tumor cells. In the innate immune arm, cells release a variety of cytokines to support the immunological activities in the tumor microenvironment. NK cells directly lyse abnormal cells. Monocytes/macrophages and dendritic cells take up debris from dead cancer cells to present peptide fragments of tumor antigens to T-cells through the major histocompatibility complex (MHC) molecules. Such antigen presentation activates the subpopulation of B and T-cells that express tumor antigen recognition receptors to proliferate and differentiate. B cells generate a humoral response by secreting antibodies specific to tumor antigens. T-cells are classified into two major subsets: CD4^+^ helper T-cells release immunomodulatory cytokines, and CD8^+^ cytolytic T-cells act as effector cells to directly lyse tumor cells during the adaptive antitumor immune response (28–31).

Thus, the immune system employs coordinated innate immunity and adaptive immunity to fight tumors. This observation provides the rationale for boosting the efficacy (including strength and precision) of an adaptive antitumor immunotherapy such as checkpoint blockade by targeting innate immune cells to activate of the adjuvant response or priming effect (28–31). TLRs are broadly expressed in immune cells for the detection of microbial pathogens to initiate host responses to infection (32–34). Synthetic TLR agonists such as imiquimod have been approved for anti-virus and cancer therapies, and others are being investigated for mono- or combination cancer therapies (10, 35–37). In the following discussion, we will focus on advances in the use of CpG-oligodeoxynucleotide (CpG-ODN), a synthetic TLR9 agonist to increase the efficacy of cancer immunotherapy with checkpoint blockade.

## TLR9 Function, Cellular Localization, and Signaling

The innate immunity is essential for host defense against microbial infections. Innate immune cells use a diverse variety of pattern recognition receptors (PRRs), including TLRs, to detect various microbial pathogen-associated molecular patterns (PAMPs). Such recognition initiates immediate innate immune responses, leading to the development of adaptive immune responses (33, 38–40). Thirteen TLRs (TLR1–13) have been identified in mammals, and ten (TLR1–10) are expressed in humans. These TLRs recognize a diverse variety of microbial PAMPs via their extracellular domain consisting of multiple leucine-rich repeats (LRRs) (41–45). TLR1, TLR2, TLR6, and TLR10 comprise a subfamily. TLR2 recognizes a broad range of microbial products, including lipoproteins, lipoteichoic acids, lipoarabinomannan, peptidoglycan, glycophosphatidylinositol anchors, zymosan, and prions. TLR2 and TLR1 form a complex that selectively recognizes bacterial lipoproteins and triacyl lipopeptides, whereas a heterodimer composed of TLR2 and TLR6 preferentially recognizes mycoplasma macrophage-activating lipopeptide 2 (46–51). The natural ligand of TLR10 is not yet well characterized; however, a recent study showed that this TLR recognizes double-stranded RNA (dsRNA) (52). TLR4 and TLR5 are closely related. TLR4 recognizes lipopolysaccharides from gram-negative bacteria, and TLR5 recognizes bacterial flagellin (53, 54). The members of the TLR3, TLR7, TLR8, and TLR9 subfamilies recognize nucleic-acid–derived structures. TLR3 detects double-stranded RNA (dsRNA) generated from viral replication in infected cells (55). TLR7 and TLR8 interact with single-stranded RNA viruses such as influenza virus and the vesicular stomatitis virus (56, 57). TLR9 responds to unmethylated CpG-DNA, including microbial DNA from DNA viruses (58, 59). In addition, TLRs recognize a wide variety of endogenous danger-associated molecular patterns (DAMPs) released from dead cells in damaged tissues. These DAMPs are cellular components and stress-induced gene products such as extracellular matrix components, extracellular proteins, intracellular proteins, and nucleic acids (60, 61).

**Figure 1:**
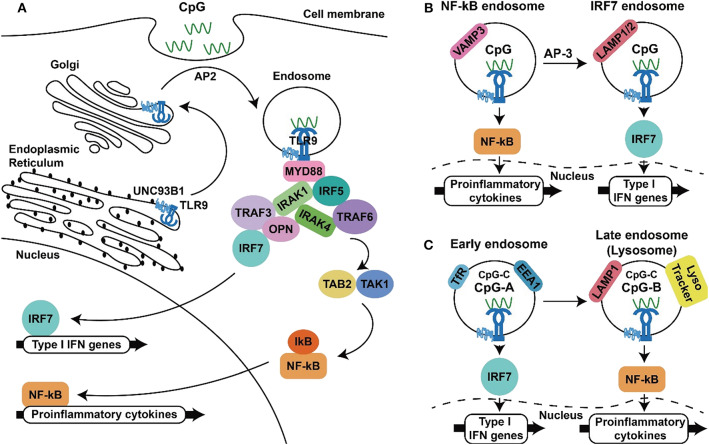
TLR9 signaling to produce inflammatory cytokines and type I IFNs. **(A)** TLR9 synthesized in the endoplasmic reticulum traffics through the ER, Golgi to endosome with the aid of UNC93B and AP2, where TLR9 interact with CpG-ODN, recruits MyD88 and downstream signaling molecules to activate NF-κB and IRF-7, resulting in the production inflammatory cytokines and type I IFNs **(B,C)**. Two proposed models for the spatiotemporal activation of NF-κB and IRF-7 at different type of endosomes. In the first model, TLR9 triggers NF-κB activation from the VAMP3^+^ endosomal compartments within minutes after activation and initiates IRF-7 activation in the LAMP1/2 endosomal compartment in 30 min to hours **(B)**. In the second model in plasmacytoid dendritic cells, class A CpG-ODNs activate IRF-7 to produce type I IFNs from the EEA1^+^, TfR^+^ early endosomes. In contrast, class B CpG-ODNs activate NF-κB for inflammatory cytokine production in LAMP1^+^ and lysoTracker^+^ late endosomes/lysosomes **(C)**. The capability of class C CpG-ODNs to activate production of type I IFNs and inflammatory cytokines is in between the capabilities of CpG-ODNs in class A and class C.

Of the TLRs, TLR9 has the narrowest cell expression profile. In humans, this TLR is constitutively expressed in B cells and plasmacytoid dendritic cells (pDCs) and to some extent is also expressed in activated neutrophils, monocytes/macrophages, cDCs, and T-cells. In addition, TLR9 has been shown to be expressed in some non-immune cells, including keratinocytes and gut, cervical, and respiratory epithelial cells (37, 62, 63). Distinct from other TLRs, TLR3, TLR7, TLR8, and TLR9 are located in intracellular vesicles (64–66). In resting cells, TLR9 is localized in the endoplasmic reticulum (ER) and must be trafficked to endosomes for activation by its agonist. The intracellular trafficking of this TLR is regulated by accessory proteins such as UNC-93 homolog B1 (UNC93B1) and specific adaptor proteins (APs). UNC93B1 interacts with TLR9 in the endoplasmic reticulum (ER) and follows the secretory pathway through the Golgi apparatus to the plasma membrane via coat protein complex II (COPII) vesicles. At the cell membrane, UNC93B1 recruits the adaptor protein AP-2 for the endocytosis of TLR9 via clathrin-containing vesicles. In the endosome, TLR9 interacts with its agonist CpG-DNA, which also enters cells via endocytosis [Figure 1A, (67–69)].

This engagement culminates in two outcomes: activation of NF-κB to produce inflammatory cytokines and activation of interferon regulatory factors (IRFs) to produce type I interferons (IFNs). Myeloid differentiation primary response 88 (MyD88) is required for TLR9 signal transduction, as MyD88 deficiency abolishes downstream signaling for cytokine productions following TLR9 activation (70). Following recruitment by TLR9, MyD88 in turn interacts with interleukin-1 receptor-associated kinase-1 (IRAK-1) and IRAK-4 through its death domain. IRAK-4 phosphorylates IRAK-1, up-regulating its kinase activity, which leads to the recruitment of tumor necrosis factor associated factor 6 (TRAF6) and the activation of transforming growth factor-β associated kinase 1 (TAK1). This cascade results in activation of the transcription factors NF-κB, which are responsible for the transcription of pro-inflammatory cytokine genes, including IL-6, IL-12 and TNF-α (71–74). Other than this, the transcription factor IRF5 is reported to be indispensable for TLR9-mediated production of pro-inflammatory cytokines. IRF5 interacts with MyD88, and TLR activation induces nuclear translocation of this transcription factor to promote gene expressions (75). In addition to inducing pro-inflammatory cytokine expression, TLR9 activation results in the production of type I IFNs, which are composed of multiple IFN-αs and a single IFN-β. These IFNs play a critical role in TLR9-mediated antitumor responses because they are involved in activation of the adaptive immune response required for tumor-cell killing (76–78). IRF-7 is a transcription factor expressed in pDCs that regulates the expression of type I IFN genes. IRF-7 associates with the complex of MyD88, IRAK1, IRAK4, and TRAF6, where IRF-7 becomes phosphorylated and translocates into the nucleus to induce transcription of type I IFNs (79, 80). In mice, TLR9-mediated production of IFNs is abrogated in cells deficient in osteopontin (Opn) and TRAF3, whereas the production of IL-12 unaffected, suggesting that Opn and TRAF3 are involved in the signaling pathway that mediates TLR9-induced activation of type I IFN production [Figure 1A, (81, 82)].

Two different mechanisms have been proposed for this signal-bifurcated process. One model suggests that TLR9 from the Golgi enters endosomes and then the VAMP3^+^ (vesicle-associated membrane protein 3) endosomes, leading to inflammatory cytokine expression. Subsequently, with the aid of AP-3, TLR9 is shuttled to LAMP1/2^+^ (lysomal associated membrane protein 1/2) lysosome-related organelles (LROs) to promote the production of type I IFNs [Figure 1B, (83, 84)]. In contrast, another model suggested that TLR9 activation, signalings leading to gene transcription of inflammatory cytokines and type I IFNs come from different type of endosomes. In this model, TLR9 activation in the TfR (transferrin receptor) and EEA1 (early endosomal antigen 1) expressed early endosomes results in IRF-7 activation and production of type IFNs, whereas activation of TLR9 in the LAMP1 and LysoTracker positive late endosome/lysosome lead to the activation of NF-κB and production of inflammatory cytokines [Figure 1C, (85–87)]. Although the location of TLR9 required to trigger such signaling is uncertain, the acidic pH of the endolysosomal compartments is thought to be required for ligand recognition of TLR9, as compounds that interfere with endosomal acidification, such as bafilomycin A1 and chloroquine, are inhibitors of TLR9 activation (88).

## Synthetic CpG-ODNS FOR TLR9 Activation

The immunostimulatory activity of microbial DNA was first observed in a DNA fraction of bacillus Calmette–Guerin (89, 90). Further studies revealed that the presence of unmethylated CpG deoxynucleotides in a particular context called the CpG motif is required for such DNA activity (91, 92). *In vivo* studies of gene knockout mice and *in vitro* studies of cell-based TLR9 activation assay later showed that TLR9 is the cellular receptor for CpG-DNA (58, 59, 93). The presence of CG dinucleotides in eukaryotic DNA is lower than in the prokaryotic DNA sequences. Further more the frequency of methylation on CpG sites are higher within eukaryotic DNA than in microbial DNA (94, 95). This difference in CpG-methylation provides a molecular base for TLR9 to distinguish self from non-self DNA in the host defense immune response to microbial infections (91, 96). Synthetic CpG-ODNs mimicking the immunostimulatory nature of microbial CpG-DNA were developed for therapeutic use (77, 96, 97). Natural microbial DNA contains a phosphodiester backbone that is easily degraded by nucleases *in vivo*. Replacement of the oxygen in the nucleic acid phosphate group with sulfur creates CpG-ODNs with a phosphorothioate backbone, making them more resistant to nucleases (98, 99).

CpG-ODNs are classified into three major classes based on their structure. The Class A CpG-ODNs (also known as type D) consist of a central phosphodiester palindromic region with one or more CpG-motifs and contain poly (G) sequences with a phosphorothioate backbone attached to both of the 5' and 3' ends. Class B (type K) CpG-ODNs contain several CpG-motifs and a phosphorothiolate backbone throughout the entire sequence. Class C CpG-ODNs contain one or two CpG-motifs, an entire phosphorothioate backbone, and a palindromic sequence at the 3' end (100–103). More recently, CpG-ODNs with different structural features have been developed to improve their effectiveness and reduce their toxicity. For example, IMO-2125 is generated by linking two CpG-ODN together through their 3' ends (104). MGN1730 contains two loops of CpG-ODN, each containing three CpG-motifs linked by a double-stranded linker (105). Another design employs CpG-ODN conjugated with an antisense oligonucleotide of signal transducer and activator of transcription (STAT3), an oncogenic transcription factor. The first generation of this CpG-STAT3 inhibitor (CSI-1) uses RNA interference for STAT3 silencing. The second generation of this molecule (CSI-2) uses a decoy oligodeoxynucleotide to increase its nuclease resistance (106, 107).

The immunostimulatory activity of a CpG-ODN is dependent on its structure. Class A CpG-ODNs induce maturation of pDCs, have little effect on B cells, and activate the production of large amounts of IFN-α. Class B CpG-ODNs strongly induce B-cell proliferation, activate pDC and monocyte maturation, NK cell activation, and inflammatory cytokine production. These CpG-ODNs also stimulate the production of IFN-α, but to a lesser extent than do the class A CpG-ODNs. The capability of class C CpG-ODNs to induce B-cell proliferation and IFN-α production is between that of class A and B CpG-ODNs (100–103). The distinct abilities of class A and class B CpG-ODNs in induction of type I IFNs is resulted from their higher order structures. Class A CpG-ODNs are able to form multimeric aggregates with a diamteter of about 50 nm. In contrast, Class B CpG-ODNs are monomeric and do not have such a feature (108). Further, a model of spatiotemporal regulation of TLR9 as shown in the Figure 1C has been suggested to explain the differential immunostimulatory activities of CpG-ODNs. According to this model, Class A CpG-ODNs activate TLR9 in early endosomes to trigger IRF7 activation, inducing the production of large amounts of IFNs. Class B CpG-ODN is quickly transported to late endosomal/lysosomal compartments for TLR9 activation to activate NF-κB and produce inflammatory cytokines. In contrast, class C CpG-ODNs can be retained in these endosomal compartments, where they activate the production of IFNs and inflammatory cytokines (85–87). In line with these, encapsidation of class B CpG-ODNs into particles allow their retention in eraly endosomes for induction of higer level of type I IFNs (109).

The structure–function relationship of class B CpG-ODNs has been extensively investigated to enable their clinical use. The immunostimulatory activity of class B CpG-ODNs depends on their nucleotide sequence, CpG-dideoxynucleotide–containing hexamer motifs (CpG motif), and the number, spacing, position, and bases surrounding these CpG-motifs (100, 110, 111). Moreover, the activity of these CpG-ODNs differs between species, a phenomenon known as “species-specific activity.” This activity of a CpG-ODN is determined by the nucleotide context of its CpG-motifs. For example, CpG-2007, which contains 22 nucleotides and three copies of the GTCGTT-hexamer motif, is more potent in activating human cells than is CpG-1826, which contains 20 nucleotides and two copies of the GACGTT-hexamer motif. In contrast, CpG-1826 is more potent in activating murine cells than is CpG-2007 (93, 100, 110–112). The nucleotide length of CpG-ODN also plays a significant role in determining its species specificity. In rabbit cells, CpG-C4609, which contains 12 nucleotides and one AACGTT-hexamer motif, generates a stronger immune response than does CpG-2007 or CpG-1826 (113).

## CpG-ODNS as Cancer Therapeutics

The activation of TLR9 by CpG-ODNs induces the immune response in two phases, innate immune and adaptive immune responses (96, 114, 115). Within hours of CpG-ODN stimulation, an antigen-independent innate immune response is elicited for an early immune response and for priming the subsequent adaptive immune responses. During this first innate immune response phase, DCs and B cells are activated. DCs are the most effective antigen-presenting cells (APCs). In addition to presenting extracellular antigens on MHC Class II molecules to CD4^+^ T-cells, DCs also mediate cross-presentation of extracellular antigens on MHC Class I molecules to CD8^+^ cytotoxic T-cells. These activities are crucial for establishing effective anti-cancer immunity (116–118). DCs produce inflammatory cytokines and type I IFNs through the activation of NF-κB and IRF. B cells produce cytokines, including IL-6 and IL-12, and chemokines via NF-κB activation. In turn, macrophages and NK cells are activated by IFNs released from pDCs. The macrophages and DCs are major IFN-γ-producing cells and APCs, and NK cells are capable of direct tumor killing during the CpG-ODN-induced antitumor response (119–122). These CpG-ODN–activated early immune responses are followed by a second phase of antigen-specific immune response that occurs several days later. B-cell stimulation by CpG-ODNs increases their sensitivity to antigen stimulation and promotes their differentiation into antibody-secreting plasma cells, increasing their production of antigen-specific antibodies (123, 124). Further, during this stage, CpG-ODN-activated APCs become competent for antigen presentation and the production of Th1-response–promoting cytokines. Increased expression of costimulatory molecules such as cluster of differentiation 80 (CD80), CD86, and molecules of the MHC increases the antigen-presenting activity of these cells to naïve T cells. The produced cytokines (TNF-α, IL-12, and IFNs) promote the T-helper-1 polarization of CD4^+^ T cells. These result in expension of antigen-specific CD8^+^ T cells (96, 114, 115, 125–127).

Because these immune responses facilitates eradication of cancer cells from bodies, the antitumor effect of CpG-ODNs has been investigated (76–78). In mouse tumor models, CpG-ODN monotherapy showed modest activity in inducing T-cell-mediated tumor regression. Injection of CpG-ODN into tumor exerted better anti-tumor activity than administration of the CpG-ODN at distant sites such as via intraperitoneal injection or intravenous injection (128, 129). Combining CpG-ODN with other therapeutics such as radiotherapy, chemotherapy, antitumor antibody, or DNA-based vaccination usually achieves greater tumor eradication (130–136). The effects resulting from combination therapy and local administration indicate that CpG-ODN exerts an adjuvant effect in the tumor microenvironment. Because tumor destruction by other therapies promotes the release of tumor antigens into the tumor microenvironment, injection of CpG-ODN into the site where tumor antigen is released has a greater effect on DC activation and antigen presentation to elicit a tumor-specific T-cell response (76–78).

Based on the positive results of preclinical studies showing that TLR9 activation can induce adjuvant effects to promote T-cell activation and reduce tumor burden, CpG-ODNs have been investigated in clinical trials as therapeutic antitumor agents (10, 35, 137, 138). The most widely investigated CpG-ODN is the B class agent PF-3512676 (also known as CpG-2006, CpG-7909, Agatolimod). Monotherapy with PF-3512676 has been investigated for treating basal cell carcinoma, renal cell cancer, melanoma, and cutaneous T-cell lymphoma via different routes, including subcutaneous, intravenous, and intratumoral injection. In patients, this CpG-ODN elicits cytokine production and antitumor T-cell responses with minimal toxicity beyond the local injection site reaction; however, its efficacy in reducing tumor growth is relatively low (139–142). Therefore, the efficacy of combination therapies using CpG-ODN with existing cancer therapeutics were investigated. In a phase II randomized trial with 184 stage IIIb/c or stage IV melanoma patients, the effect of subcutaneous PF-3512676 in monotherapy and combination therapy with intravenous dacarbazine (DITC) was investigated. Patients received either 10 mg of PF-3512676, 40 mg of PF-3512676, 40 mg of PF-3512676 plus DITC, or DITC alone as a control. The object response rate (ORR) was greatest in patients treated with 40 mg of PF-3512676 plus DITC. Nevertheless, no significant difference in overall survival (OS) or median time to progression was observed between treatment groups. Thus, the phase III portion of this study was not continued (143). Another randomized phase II trial evaluated the activity of subcutaneous PF-3512676 in combination with first-line taxine/platinum chemotherapy in 111 patients with non-small-cell lung cancer. The ORR (confirmed and unconfirmed) was 38% in the PF-3512676 arm (*n* = 74) and 19% in the chemotherapy-alone arm (*n* = 37). The median survival was 12.3 months in the PF-3512676 arm and 6.8 months in the chemotherapy-alone arm, with one-year survival of 50 and 33%, respectively (144). The combination of PF-3512676 with standard chemotherapy was further evaluated as a first-line treatment for advanced non–small-cell lung cancer in phase III trials. In one trial with 828 patients, the combination of subcutaneous PF-3512676 with intravenous paclitaxel/carplatin was compared with paclitaxel/carplatin alone. No significance improvement in OS or progression-free survival (PFS) was observed for PF-3512676 combination therapy. In another trials, comparison of PF-3512676 combined with gemcitabine/cisplatin and gemcitabine/cisplatin alone revealed a similar median OS and PFS in these two treatments (145, 146). To date, no CpG-ODN has been approved for cancer treatment, but a wide variety of clinical studies exploring the potential of CpG-ODNs including in combinational use with immune checkpoint inhibitors for cancer therapy are still ongoing (138, 147).

## Combination Therapy With CpG-ODNS and Immune Checkpoint Inhibitors

Immune checkpoints are regulators of the immune system that maintain the immune response in a normal physiologic range and prevent inflammatory or autoimmune disorders resulting from over-activation of immune system. CTLA-4 and PD-1 are the two best-characterized immune checkpoint regulators (148–151). The expression of CTLA-4 is upregulated immediately following engagement of the T-cell receptor. This protein competes with the costimulatory receptor CD28 for its B7 ligands, CD80 (B7-1) and CD86 (B7-2), thereby interfering with the activation of CD28-mediated costimulatory signaling by these two ligands and attenuating T-cell activation. Because the negative regulatory function of CTLA-4 involves the expression of B7 ligands and CD28 signaling, CTLA-4 limits the early immune responses of T cells in lymphoid tissue (13, 152–154). In addition, CTLA-4 attenuates T-cell activation in peripheral tissues, as B7 ligands are constitutively expressed at differing levels in APCs and activated T cells. These observations suggest that CTLA-4 plays a central role in the regulation of T-cell activation and is critical for immune tolerance (14, 15, 149). In contrast to CTLA-4, PD-1 is expressed in activated and also exhausted T cells, B cells, and myeloid cells (16, 155, 156). Two ligands of PD-1, PD-L1 and PD-L2 are identified. Of them, PD-L2 induces IL-12 production in DCs. Given that IL-12 is important for T-cell differentiation into Th1-type cells, PD-L1 is a better target for inhibition to elicit antitumor immune response than is PD-L2 (18, 19, 157). PD-1 mainly regulates the late immune response of T cells in peripheral tissues, as its ligands are widely expressed in non-lymphoid tissues. Engagement of PD-1 with its ligand negatively regulates T-cell activation by activating the tyrosine phosphatase SHP2, which dephosphorylates and inactivates molecules involved in TCR signaling. SHP2 was also shown to regulate CD28 signaling through its phosphatase activity (17, 149, 158–160). These observations suggest that CTLA-4 and PD-1 regulate T-cell activation by distinct but somewhat overlapping molecular mechanisms (148–151).

Because CTLA-4 and PD-1 act through ligand-receptor interactions, their activity can be blocked by specific monoclonal antibodies. Indeed, a variety of CTLA-4 and PD-1/PD-L1 monoclonal antibodies have been developed for immune checkpoint blockade. The anti-CTLA-4 antibody ipilimumab was approved by the FDA in 2011 for treating metastatic melanoma. Since then, six additional antibodies targeting PD-1 or PD-L1 have been approved for immunotherapy of different cancer types (Table 1). These checkpoint blockade therapies demonstrate notable efficacy for cancer treatment, nevertheless a large fraction of patients still fails to response to this treatment, indicating a tremendous need to improve the efficacy of therapies employing immune checkpoint inhibitors (149, 186, 187). The resistance of patients to immune checkpoint therapies may be caused by deficiencies in various aspects of T-cell activation for the antitumor response. Possibilities include poor immunogenicity of the tumor resulting from insufficient formation of tumor antigen and antigen presentation, inadequate T-cell activation and killing activity, and altered T-cell trafficking. Therefore, combining an immune checkpoint inhibitor with other treatment may increase the efficacy of such therapies (188–192).

**Table 1:** FDA-approved antibodies targeting immune checkpoints for treating different type of cancers.

**Inhibitor**	**Target**	**Approved**	**Tumor type**	**References**
Ipilimumab (Yervoy®)	CTLA-4	2011	Advanced melanoma	(1)
		2018	Metastatic RCC (in combination of nivolumab), and CRC	(161, 162)
Pembrolizumab (Keytruda®)	PD-1	2014	Advanced melanoma	(163)
		2015	Metastatic NSCLC	(164)
		2016	Head and neck cancer	(165)
		2017	Classical Hodgkin lymphoma, urothelial carcinoma, any solid tumor with a specific genetic feature, and advanced gastric and gastroesophageal junction adenocarcinoma	(166–169)
		2018	Advanced cervical cancer, and HCC	(170, 171)
		2019	Advanced RCC (in combination of axitinib)	(172)
Nivolumab (Opdivo®)	PD-1	2014	Advanced melanoma	(173)
		2015	Lung cancer, and metastatic RCC	(174, 175)
		2016	Hodgkin lymphoma, and head and neck cancer	(175, 176)
		2017	Advanced urothelial carcinoma, CRC, and HCC (previously treated with sorafenib)	(177, 178)
Atezolizumab (Tecentriq®)	PD-L1	2016	Advanced urothelial carcinoma, and NSCLC progressed in platinum-containing therapy	(175, 179)
		2018	Advanced bladder cancer	(180)
		2019	PD-L1 positive TNBC (in combination of abraxane), and SCLC (in combination of carboplatin and etoposide)	(181, 182)
Avelumab (Bavencio®)	PD-L1	2017	Merkel cell carcinoma, and urothelial cancer	(167, 183)
		2019	Genitourinary cancer	(172)
Durvalumab (Imfinzi®)	PD-L1	2017	Advanced urothelial cancer	(167)
		2018	NSCLC	(184)
Cemiplimab-rwlc (Libtayo®)	PD-1	2018	Advanced cutaneous squamous cell carcinoma	(185)

*RCC, renal cell carcinoma; CRC, colorectal cancer; NSCLC, non-small cell lung cancer; HCC, hepatocellular carcinoma; TNBC, triple-negative breast cancer; SCLC, small cell lung cancer*.

**Figure 2:**
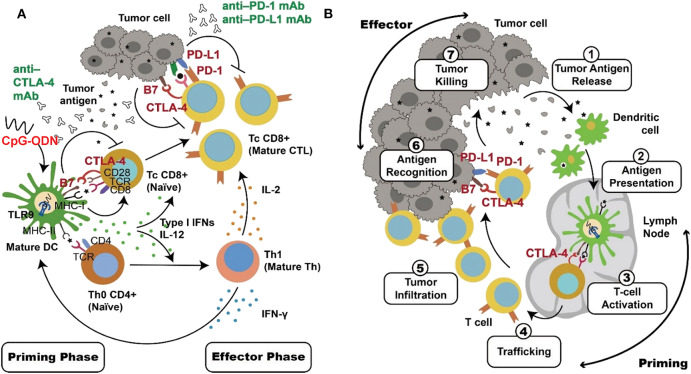
Complementary mechanisms of TLR9 activation and checkpoint blockade in combinational cancer immunotherapy. DCs and T cells play key roles in the antitumor immune response. These two types of cells are major target for TLR9 agonists and immune checkpoint inhibitors, respectively. **(A)** Activation of TLR9 by CpG-ODN triggers innate immune responses, including cytokine production and the uptake and presentation of tumor antigen in DCs. These adjuvant effects, particularly the production of IL-12 and type I IFNs, facilitate a Th1 response of T cells and expansion of tumor-specific T cells during the priming phase. Immune checkpoint blockade by anti-PD-1/anti-PD-L1 antibody release inhibition of CD8^+^ cytotoxic T-cell activation during the effector phase. In contrast, anti-CTLA-4 inhibition activates T cells during both of the priming and effector phases. These events lead to a more effective and more specific adaptive immune response for tumor-cell killing. **(B)** DCs and T cells involved in the antitumor immune response serve different immunological functions in different locations, as illustrated.

A process of T-cell mediated antitumor response includes a priming phase which mainly involves with innate immune responses and an effector phase of an adaptive immunological tumor killing by T cells as shown in Figure 2. In the priming phase, activated APCs, such as dendritic cells, produce IL-12 and type 1 IFNs to facilitate a CD4+ T-cell-mediated Th 1 response. In addition, the dendritic cells produce costimulatory molecules and present antigen from a patient's cancer cells to promote proliferation of tumor-specific cytotoxic CD8+ T cells. These T cells then migrate to tumor sites, displaying their tumor-killing effects during the effector phase (29–31, 193–195). According to this mechanism, combination therapy including a TLR9 agonist and immune inhibitor is promising because these two agents use different and complementary mechanisms to up-regulate the T-cell-mediated antitumor response (138, 189–192). Activation of TLR9 in dendritic cells by CpG-ODN initiates the immune response via production of the costimulatory molecules CD80 and CD86 and cytokines TNF-α, IL-6, IL-12, and type I IFNs. Moreover, injection of CpG-ODN into the tumor site can induce local tumor-cell death, releasing more tumor antigens into the tumor microenvironment and activating antigen uptake and presentation by dendritic cells. These events promote effective generation of tumor-specific cytotoxic CD8^+^ T cells during the priming phase (86, 87, 120, 122). In contrast, the immune checkpoint inhibitors release the inhibition of T-cell activity to promote tumor-cell killing during the effector phase (148–151). Thus, cancer therapy using a combination of TLR9 activation and immune checkpoint blockade can result in more robust and more specific tumor killing (Figure 2).

Studies using melanoma mouse models have shown that a synergistic effect on tumor regression results from combining CpG-ODN-mediated activation of APCs with immune checkpoint inhibitor-mediated T-cell activation (136, 196). Similar synergy resulting in longer survival was also observed in murine bladder cancer when CpG-ODN was combined with CTLA-4 or PD-1 inhibitors (197). Another studies revealed that CpG-ODN can revert resistence to PD-1 blockade therapy by expending CD8+ T cells in colon cancer animal model, enhances the efficacy of anti-PD-1 in head and neck cancer animal model (198, 199). CpG-ODN modulates tumor microenvironment, turns “cold” tumor into “hot” tumor, enhances the anti-tumor effect of immune checkpoint blockade in colon cancer animal model (200). Moreover, CpG-ODN delivered by inhalation is capable of priming T-cell responses against a poorly immunogenic lung tumor (201). The encouraging results in these animal studies provided the rationale for combined clinical regimens using CpG-ODNs and immune checkpoint inhibitors simultaneously. Several clinical investigations of such combination therapy are presently underway. CMP-001 is a class A CpG-ODN encapsulated into virus-like particles to render it stable. In a study of 69 patients with advanced melanoma and resistance to pembrolizumab therapy, CMP-001 and pembrolizumab were directly injected into the accessible lesions. The response rate was 21.7%, and an abscopal effect was observed, with shrinkage occurring in non-injected cutaneous, nodal, hepatic, and splenic metastases (202). In a study of SD-101/pembrolizumab combination therapy in 9 advanced melanoma patients naïve to anti-PD-1 therapy, a response rate of 78% was observed (203). Similar to the study of CMP-001, the SD101 exerted an abscopal effect, with tumor shrinkage observed in both the injected and non-injected lesions (202, 203). A clinical study of IMO-212 (Tilsotolimod), another TLR9 agonist, was conducted in a cohort of 26 patients with PD-1-inhibitor– refractory advanced melanoma. Combined therapy with IMO-2125 and ipilimumab resulted in an ORR of 38.1%, an increase over the 13% reported in a previous study of ipilimumab treatment alone. The disease control rate was 71.4% for the combination therapy, and an abscopal effect was observed with no synergistic toxicity. A global phase III randomized study comparing IMO-2125 plus ipilimumab to ipilimumab alone for treating PD-1-inhibitor refractory cancer is underway (204, 205). Combination therapy using TLR9 agonists and different immune checkpoint inhibitor are under clinical investigation for treating melanoma and other types of tumors (Table 2).

**Table 2:** Current clinical trials of combination cancer immunotherapies using a TLR9 activation agonist and a checkpoint blockade agent.

**TLR9 agonist**	**Class**	**Phase**	**Treatment**	**In combination with**	**Tumor type**	**References**
CMP-001	A	II	I.V.	Nivolumab	Melanoma and lymph node cancer	NCT03618641
		I	I.T.	Pembrolizumab	Melanoma	NCT02680184
		I	S.C.	Ipilimumab, and nivolumab	Metastatic CRC	NCT03507699
		I/II	I.T.	Avelumab	SCCHN	NCT02554812
		I	IT/SC	Atezolizumab	NSCLC	NCT03438318
IMO-2125 (Tilsotolimod)		III	I.T.	Ipilimumab	Anti-PD-1 refractory melanoma	NCT03445533
		II	I.T.	Ipilimumab, and nivolumab	Solid tumors	NCT03865082
		I/II	I.T.	Ipilimumab, or pembrolizumab	Metastatic melanoma	NCT02644967
MGN1703 (Lefitolimod)		I	S.C.	Ipilimumab	Advanced cancers	NCT02668770
SD-101 (Dynavax)	C	II	I.T.	Pembrolizumab	Prostate cancer	NCT03007732
		I	I.T.	Nivolumab	Chemotherapy-refractory metastatic pancreatic adenocarcinoma	NCT04050085
		I/II	I.T.	Pembrolizumab	Metastatic melanoma or recurrent or metastatic HNSCC	NCT02521870
AST-008		I/II	I.T.	Pembrolizumab	Advanced solid tumors	NCT03684785
DV281	C	I	Inhaled	Nivolumab	Advanced NSCLC	NCT03326752

*I.T., Intratumoural; I.V., Intravenous; S.C., Subcutaneous; CRC, colorectal cancer; SCCHN, squamous cell carcinoma of head and neck; NSCLC, non-small cell lung cancer; HNSCC, head and neck squamous cell carcinoma*.

## Conclusion and Perspectives

The field of cancer immunotherapy has progressed significantly since the approval of ipilimumab in 2011. Therapy with immune checkpoint blockade has revealed benefits to cancer patients, improving their survival and quality of life. Despite breakthroughs in the field, the pool of patients benefiting from this therapy is relatively small. Thus, investigating combinations of immune checkpoint inhibitors with other currently available or novel cancer therapeutics is needed to maximize the benefits of this cancer immunotherapy. Activation of TLR9 by CpG-ODN elicits the antitumor immune response. A wide variety of clinical trials are presently investigating the use of CpG-ODNs in antitumor therapy. Although no CpG-ODN has been approved for use as a cancer therapeutic agent, one such agent (CpG-1018) is used as an adjuvant in a Hepatitis B vaccine (HEPLISAV-B) approved by the FDA in 2018. This vaccine is proven more effective against Hepatitis B than those using aluminum salt as the adjuvant (206, 207). This observation suggests that CpG-ODN is a potent adjuvant and that is safe for therapeutic use. The mechanism of action of CpG-ODNs in activating the antitumor immune response is distinct and complementary to that underlying immune checkpoint blockade. Thus, the rationale for combining these agents for cancer therapy is sound. A number of clinical trials of therapies combining these two agents are presently underway for a variety of cancer types. The results will reveal whether combining these agents improves the efficacy of cancer immunotherapy using immune checkpoint inhibitors. Worth to note, although this review is focused on the antitumor effect of TLR9 activation, agonists of other TLRs were also shown to have antitumor activities. Imiquimod, a TLR7 agonist had FDA approved for treatment of superficial basal cell carcinoma in 2004 (3–5). Others including CADI-05 (TLR2 agonist), BO-112 (TLR3 agonist) and G100 (TLR4 agonist) were investigated in clinical trials for their antitumor effects (208–210). Whether these TLR agonists can improve the efficacy of immune checkpoint inhibitors in combinational therapies is also received attention.

## Author Contributions

All authors were involved in researching data for this article, contributed to discussion of the content, preparing, and writing the manuscript.

## Conflict of Interest

The authors declare that the research was conducted in the absence of any commercial or financial relationships that could be construed as a potential conflict of interest.
